# Rab8 attenuates Wnt signaling and is required for mesenchymal differentiation into adipocytes

**DOI:** 10.1016/j.jbc.2021.100488

**Published:** 2021-03-01

**Authors:** Ewa Stypulkowski, Qiang Feng, Ivor Joseph, Victoria Farrell, Juan Flores, Shiyan Yu, Ryotaro Sakamori, Jiaxin Sun, Sheila Bandyopadhyay, Soumyashree Das, Radek Dobrowolski, Edward M. Bonder, Miao-Hsueh Chen, Nan Gao

**Affiliations:** 1 Department of Biological Sciences, Rutgers University, Newark, New Jersey, USA; 2 Department of Pediatrics, Baylor College of Medicine, Children's Nutrition Research Center, Houston, Texas, USA; 3 Rutgers Cancer Institute of New Jersey, New Brunswick, New Jersey, USA

**Keywords:** Rab8, MEF, mouse embryonic fibroblast, Wnt, adipocyte, primary cilia, frizzled, differentiation, C/EBPs, CCAAT/enhancer binding proteins, FBS, fetal bovine serum, IFT, intraflagellar transport, KD, knockdown, MEF, mouse embryonic fibroblast, pLRP6, phosphorylated LRP6, PPAR-γ, peroxisome proliferator-activated receptor γ, Smo, Smoothened

## Abstract

Differentiation of mesenchymal stem cells into adipocyte requires coordination of external stimuli and depends upon the functionality of the primary cilium. The Rab8 small GTPases are regulators of intracellular transport of membrane-bound structural and signaling cargo. However, the physiological contribution of the intrinsic trafficking network controlled by Rab8 to mesenchymal tissue differentiation has not been fully defined *in vivo* and in primary tissue cultures. Here, we show that mouse embryonic fibroblasts (MEFs) lacking Rab8 have severely impaired adipocyte differentiation *in vivo* and *ex vivo*. Immunofluorescent localization and biochemical analyses of Rab8a-deficient, Rab8b-deficient, and Rab8a and Rab8b double-deficient MEFs revealed that Rab8 controls the Lrp6 vesicular compartment, clearance of basal signalosome, traffic of frizzled two receptor, and thereby a proper attenuation of Wnt signaling in differentiating MEFs. Upon induction of adipogenesis program, Rab8a- and Rab8b-deficient MEFs exhibited severely defective lipid-droplet formation and abnormal cilia morphology, despite overall intact cilia growth and ciliary cargo transport. Our results suggest that intracellular Rab8 traffic regulates induction of adipogenesis *via* proper positioning of Wnt receptors for signaling control in mesenchymal cells.

Adipocytes are differentiated mesenchymal cells that store excess food energy as fat in organelles known as lipid droplets. Metabolic disorders such as obesity disrupt lipid homeostasis and adipogenesis by inducing hypertrophy of existing adipocytes or by increasing proliferation to form new adipocytes to cope with excess fat (1, 2). Adipogenesis starts with the specification of mesenchymal stem cells into committed preadipocytes that go on to terminally differentiate into adipocytes (3, 4, 5, 6). Aberrant induction of adipogenesis may be a factor in developing obesity. It is thus imperative to fully understand intracellular and intercellular pathways that regulate adipogenesis.

A hallmark of adipocyte maturation is the formation, trafficking, and fusion of lipid droplets, all processes linked to the Rab family of small GTPases (7). Approximately 30 Rab proteins have been shown to be required for lipid droplet formation and fusion (7, 8). The two Rab8 mammalian isoforms, Rab8a and Rab8b, are regulators of anterograde membrane trafficking (9, 10), and they function in regulating cell shape, migration, apical and basolateral trafficking, and docking of secretory vesicles to the plasma membrane (11, 12, 13, 14, 15). Rab8 activity is regulated by the guanine nucleotide-exchange factor, Rabin8, and GTPase-activating proteins (14, 16). During cilia formation, Rab8 is localized to the primary cilium and may direct vesicle docking and fusion to the cilium base (13, 17). As a polarized trafficking regulator, the movement of proteins up the primary cilium may also depend on Rab8 (11, 13, 14, 17, 18, 19, 20). Rab8 also interacts with key ciliary regulators such as the BBSome (17) and intraflagellar transport (IFT) particles to traffic cargoes within the cilium and may regulate the length of the cilium (13, 21). Rab8’s role in adipogenesis has not been fully defined *in vivo* or in primary MEFs.

Adipocyte differentiation may be coordinated by Wnt, Hedgehog, bone morphogenetic protein, and hormone (namely insulin and leptin) signaling (3, 4, 5, 6). The Wnt pathway drives adipocyte differentiation by inducing transcription of genes necessary for adipogenic cell fate, such as CCAAT/enhancer binding proteins (C/EBPs) and peroxisome proliferator-activated receptor γ (PPAR-γ) (1, 3, 22, 23). A second component of adipocyte differentiation is the transient induction of the primary cilium and increased expression levels of ciliary proteins followed by its disappearance during adipocyte maturation (3, 5, 24).

Primary cilia, or nonmotile cilia, are microtubule structures at the plasma membrane made up of an axoneme arranged in 9 + 0 formation and a basal body (5, 25, 26). The primary cilium is believed to function as a hub for transducing extracellular signals, as frizzled receptors and the Hedgehog pathway component, Smoothened (Smo), are localized to its surface (3, 5, 11, 25, 26, 27, 28, 29, 30). Partial or complete impairment of cilia formation results in ciliopathies such as Bardet–Biedl Syndrome, Alström disease, and potentially obesity (2, 3, 17, 18, 25, 27, 29, 31, 32), which are accompanied with altered Wnt and Hedgehog signaling (3, 28, 33). Proteins integral to cilia assembly and function, such as the BBSome, IFT particles, and Kif3a, are expressed in preadipocytes and upregulated when preadipocytes are induced to differentiate (2, 11, 16, 17, 21, 26, 28, 33, 34, 35). Primary cilia assembly and function is also necessary to upregulate the expression of proadipogenic factors C/EBPs and PPAR-γ during adipogenesis (3, 5, 26, 36). Impaired primary cilia may prevent preadipocytes from differentiating, whereas existing mature adipocytes display increased fat accumulation (3, 36). The primary cilium may also function in inhibiting Wnt signaling by promoting β-catenin degradation (2, 25, 27).

Wnt proteins are secreted extracellular growth factors that bind the coreceptors LRP5/6 and Fzd (37, 38). Upon ligand binding, the downstream effectors Axin, CK1, and Dvl are recruited, forming a ternary complex at the plasma membrane that stabilizes cytosolic β-catenin. Once stable, β-catenin translocates to the nucleus, where it binds its transcription cofactor TCF/Lef and activates the transcription of genes necessary for the progenitor fate, cell growth, and proliferation. In the absence of Wnt ligands, β-catenin is phosphorylated by GSK3β and targeted to proteasomes for degradation by a destruction complex consisting of the E3 ubiquitin ligase β-Trcp, Axin, GSK3β, and APC (39). A fundamental Wnt-stimulated cellular response is the rapid assembly and trafficking of multiprotein intracellular plasma membrane associated complex known as the LRP6 signalosome (40). These signalosomes are marked by elevated levels of phosphorylated LRP6 (pLRP6) that recruit key Wnt components, such as Axin and Dvl, to inactivate the β-catenin destruction complex (40). Previously, we reported that Rab8a regulates Wnt secretion and maturation of Paneth cells in the mouse intestine (41) and documented the contribution of Rab and Rho GTPases to epithelial tissue homeostasis and regeneration (39, 41, 42, 43, 44, 45, 46, 47, 48, 49, 50, 51, 52, 53).

Whether Rab8-mediated membrane transport affects mesenchymal cell differentiation remains incompletely defined. We show here that MEFs deficient in Rab8a, Rab8b, or both proteins exhibit a severe impairment in activating the adipogenesis program. Mechanistically, Rab8 deficiency alters the intracellular Lrp6 vesicular compartment, Fzd2 membrane trafficking, and Wnt signaling.

## Results

### Rab8a-deficient MEFs show an increased cellular response to Wnt stimulation

**Figure 1:**
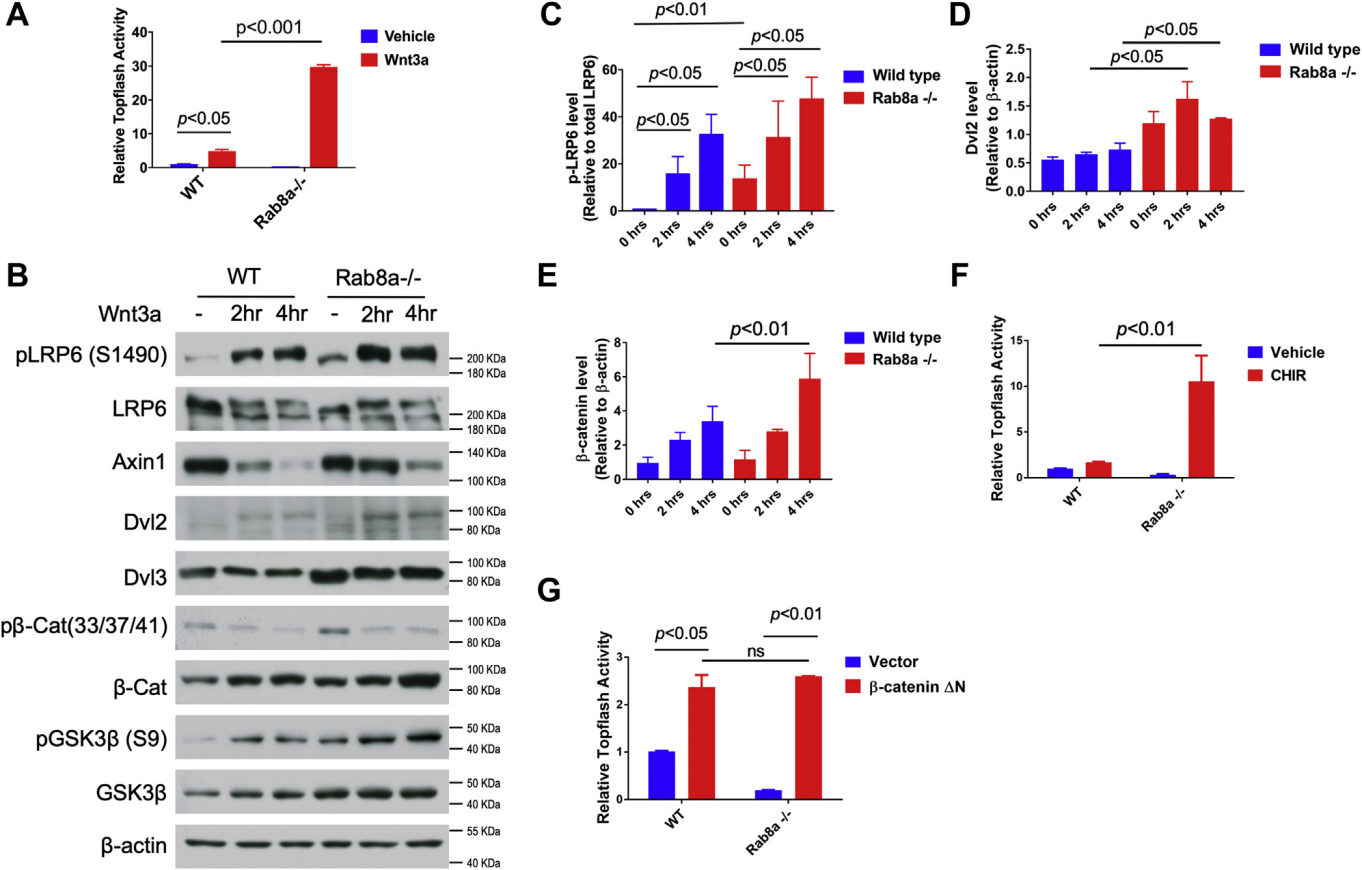
**Rab8a-deficient MEFs had an increased signaling response to Wnt stimulation.***A*, quantifications of TopFlash luciferase activities in serum-starved WT and Rab8a^−/−^ MEFs stimulated with Wnt3a (*red bar*) or vehicle (*blue bar*). *B*, western blots for Wnt pathway components were performed on whole-cell lysates of WT and Rab8a^−/−^ MEFs at 0, 2, and 4 h of Wnt3a treatment. β-Actin was used as a loading control. *C*–*E*, pLrp6, Dvl2, and total β-catenin were quantified from four independent experiments. *F*, WT or Rab8a^−/−^ MEFs were treated with the GSK3β inhibitor CHIR99021 (*red bars*) or vehicle (*blue bars*). TopFlash luciferase activities were quantified. *G*, WT or Rab8a^−/−^ MEFs were transfected with a constitutive active truncated β-catenin (β-catenin ΔN; *red bars*) or empty vector as a control (*blue bars*). TopFlash luciferase activities were quantified. *p* values were determined by Student's *t* test. *Error bars* indicate the SEM. MEFs, mouse embryonic fibroblasts.

We first investigated the role of Rab8 on induction and activation of Wnt/β-catenin signaling components in Rab8a^−/−^ MEFs. Using the TopFlash luciferase reporter as a readout for Wnt/β-catenin transcriptional activity, Rab8a^−/−^ MEFs displayed increased sensitivity to Wnt3a stimulation (Fig. 1*A*, data represent six independent experiments). WT MEFs exhibited a 4.9-fold Wnt3a-induced TopFlash activity, whereas Rab8a^−/−^ MEFs exhibited a 30-fold induction of reporter activity.

Wnt engagement with surface receptors on ligand-receiving cells elicits rapid formation of LRP6 signalosomes (40), which are biochemically characterized by a surge of cellular pLRP6 levels due to phosphorylation of LRP6’s intracellular domain by CK1γ and GSK3β (54, 55). We next tested if Rab8a affects the temporal induction of pLRP6 in serum-starved cells after Wnt3a stimulation. In untreated (serum-starved) Rab8a^−/−^ MEFs, the abundance of pLRP6 (normalized relative to the total LRP6) was approximately 14-fold of that in WT MEFs (Fig. 1, *B* and *C*). Upon Wnt3a treatment, pLRP6 levels progressively increased over 4 h after stimulation in both WT and Rab8a^−/−^ MEFs (Fig. 1, *B* and *C*, data represent four independent experiments).

Wnt-induced Lrp6 signalosomes recruit key pathway components (*e.g.*, Axin and Dvl) and promote inactivation of the destruction complex (40). The observed increase in pLRP6 in Rab8a^−/−^ MEFs was accompanied by a concomitant elevation of Dvl2, pGSK3β, and total β-catenin (Fig. 1, *B*, *D* and *E*) but not Axin1 and phosphorylated β-catenin.

These data suggested that loss of Rab8a in MEFs appeared to elevate Lrp6 signalosome activity that was accompanied by an enhanced intracellular signaling response. To examine at what molecular level Rab8a deficiency enhanced the signaling cascade, GSK3-mediated signaling inhibition was first assessed by using CHIR99021, the GSK3 inhibitor (56). Rab8a^−/−^ MEFs exhibited 9-fold increase in TopFlash activity in response to CHIR99021 compared with WT MEFs (Fig. 1*E*). We then overexpressed a constitutively active β-catenin (β-cat-ΔN) construct lacking the GSK3β phosphorylation sites (57) and found an equivalent induction of TopFlash activity in WT and Rab8a^−/−^ MEFs (Fig. 1*F*). These data suggested that the enhanced Wnt signaling in Rab8a^−/−^ MEFs occurred upstream of β-catenin, possibly at the cell surface and endocytic compartment.

### Rab8 deficiency affects LRP6 signalosome

**Figure 2:**
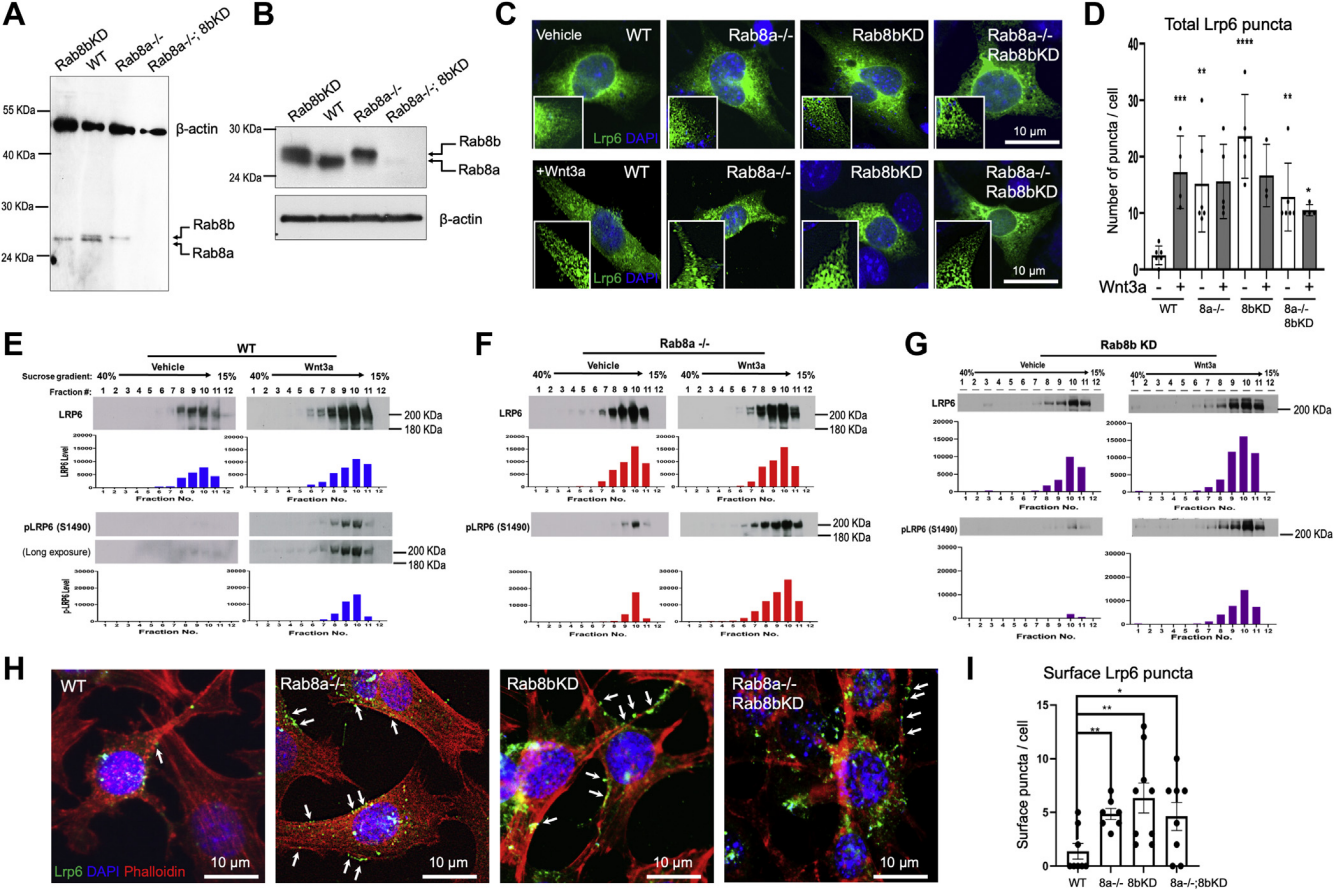
**Loss of Rab8a led to an elevated pLrp6 and an altered vesicular Lrp6 compartment.***A*, western blot showed Rab8b knockdown efficiency in Rab8b^KD^ and Rab8a^−/−^;Rab8b^KD^ MEFs. Note Rab8a and Rab8b are similar in size but could be distinguished as two isoform-specific bands. *B*, western blot using lysates of MEFs after 16 passages showed continued Rab8b KD. β-Actin was used as a loading control. *C*, serum-starved WT, Rab8a^−/−^, Rab8b^KD^, and Rab8a^−/−^;Rab8b^KD^ MEFs were transiently transfected with 0.5-μg pCS2–LRP6 GFP for 16 h. Cells were then stimulated by vehicle (Dulbecco's modified Eagle's medium) or Wnt3a for 15 min. Indirect immunofluorescence for GFP was performed to locate LRP6. Note that LRP6 puncta were rare in vehicle-treated WT MEFs but became prominent in Wnt3a-stimulated cells. *D*, the numbers of LRP6 puncta were manually counted from individual cells for each condition. Experiments were repeated five times. ∗*p* < 0.05; ∗∗*p* < 0.01; ∗∗∗*p* < 0.001 when compared with vehicle-treated WT. *E*–*G*, sucrose density cell fractionation assays were performed on serum-starved WT, Rab8a^−/−^, or Rab8b^KD^ MEFs stimulated with Wnt3a or vehicle. Western blots for total Lpr6 or pLrp6 were performed. Longer exposure of pLrp6 blot for WT in panel *E* showed minimal but detectable signal in unstimulated condition. These data represent at least three independent experiments. *H* and *I*, serum-starved WT, Rab8a^−/−^, Rab8b^KD^, and Rab8a^−/−^;Rab8b^KD^ MEFs were transiently transfected with pCS2–LRP6 GFP for 24 h. Cells were then stained for LRP6-GFP and phalloidin. The numbers of peripheral LRP6 puncta (*arrows*), based on phalloidin staining, were manually counted for individual cells of designated genotypes. ∗*p* < 0.05; ∗∗*p* < 0.01; ∗∗∗*p* < 0.001, when compared with WT. MEFs, mouse embryonic fibroblasts.

The two mammalian Rab8 isoforms, Rab8a and Rab8b, share 83% amino acid sequence homology and both participate in apical cargo transport (58). To examine whether Rab8 regulates Lrp6 vesicular compartment and MEF’s signaling capacity in response to Wnt ligands, we first established stable Rab8b knockdown (KD) MEFs and an MEF line deficient in both Rab8a and Rab8b (Fig. 2*A*). Rab8b KD efficacy was verified by Western blot even after multiple passages (Fig. 2*B*). We first examined Lrp6 intracellular localization in WT and Rab8-deficient MEFs in the presence or absence of exogenous Wnt ligands, by transiently expressing a GFP–Lrp6. In serum-starved WT MEFs treated by the vehicle, vesicular Lrp6 was rare, with approximately two Lrp6^+^ puncta per cell on average. Upon Wnt3a addition for 15 min, vesicular Lrp6 increased by approximately 9-fold (Fig. 2, *C* and *D*). In contrast, vehicle-treated Rab8a^−/−^, Rab8b^KD^, and Rab8a^−/−^;Rab8b^KD^ MEFs had significantly increased vesicular Lrp6 compartments compared with WTs, and the total numbers of Lrp6+ puncta did not increase further upon Wnt3a addition (Fig. 2, *C* and *D*).

The above results suggested a changed basal Lrp6 compartmentalization in Rab8-deficient MEFs. To biochemically examine Lrp6 intracellular distribution, we performed sucrose gradient sedimentation on lysates of MEFs upon 4 h of Wnt3a or vehicle treatments, essentially following a previous study (40). In vehicle-treated WT MEFs, Lrp6 was primarily found in fractions 8 to 10, and pLRP6 was barely detected in any of the fractions even after a longer exposure (Fig. 2*E*, data represent three independent experiments). In Wnt3a-stimulated WT MEFs, Lrp6 was detected in fractions 6 to 11, while pLrp6 became detectable in fractions 8 to 11, where Gsk3, endocytic markers, and caveolin were found to be cosedimented (Fig. S1*A*).

Vehicle-treated Rab8a^−/−^ MEFs showed notably higher basal pLrp6 in fractions 9 to 11 (Fig. 2*F*), where Dvl2, Rab7, Rab9, and caveolin were cosedimented (Fig. S1*B*). Wnt stimulation increased pLrp6 abundance and intracellular domains in Rab8a^−/−^ MEFs (Fig. 2*F*). In addition, Wnt-induced sedimentations of Axin1, Rab7, and caveolin were altered in Rab8a^−/−^ MEFs compared with WT MEFs (Fig. S1*C*), suggesting that ablation of Rab8a vesicular compartment affected the membrane trafficking network in response to exogenous Wnt ligand stimulation.

Examination of Rab8b^KD^ MEFs reached a similar but less robust effect on pLrp6, as Rab8a^−/−^ MEFs (Fig. 2*G*). Owing to the detection of basal pLrp6 in these MEFs, we examined localization of Lrp6 puncta in starved cells and found that in addition to intracellular puncta, there were significantly increased numbers of peripheral Lrp6^+^ puncta in Rab8-deficient MEFs (Fig. 2, *H* and *I*, data represent six independent experiments). These results collectively suggested that there was an altered Lrp6 vesicular compartment that potentially contributed to a sensitized MEF response to Wnt ligands.

### Loss of Rab8 impairs adipocyte differentiation

**Figure 3:**
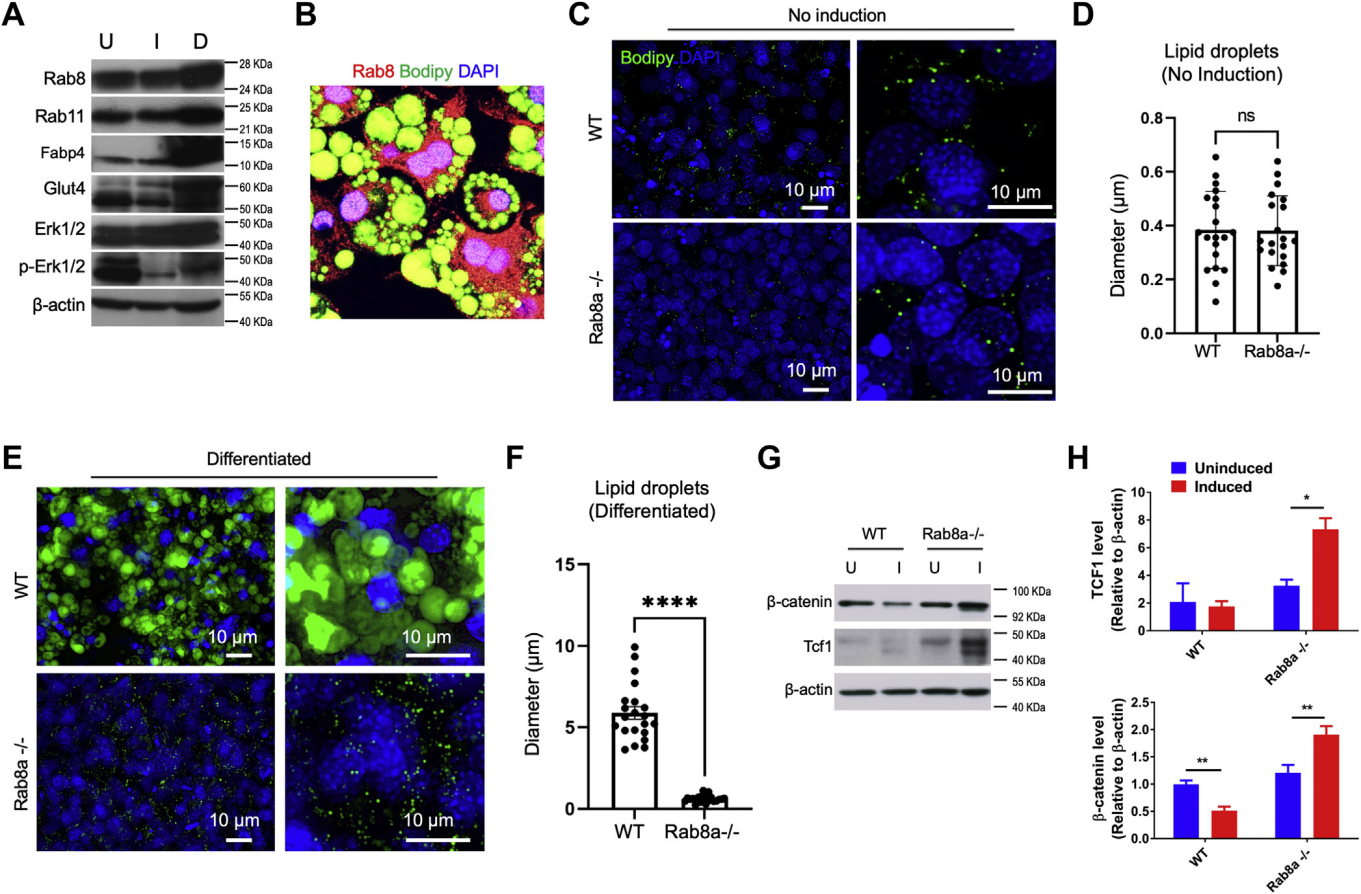
**Impaired adipocyte differentiation from Rab8a**^**−/−**^**MEFs.***A*, western blots for Rab8, Rab11, Glut4, Fabp4, and ERK1/2 were performed on total lysates prepared from uninduced WT MEFs and induced WT preadipocytes 48 h after adipogenic induction, and differentiated WT adipocytes 10 days after induction. β-Actin was used as a loading control. *B*, representative immunofluorescence of mature WT adipocytes stained for lipid droplets (BODIPY 493/503; *green*), Rab8 (*red*), and nuclei (*blue*). *C*, representative images of lipid droplets (BODIPY 493/503; *green*) in WT or Rab8a^−/−^ MEFs before induction. Nuclei were stained by DAPI in *blue*. *D*, quantification of the lipid-droplet diameter in uninduced WT and Rab8a^−/−^ MEFs. *E*, representative images of lipid-droplet staining in postinduction WT or Rab8a^−/−^ MEFs. *F*, lipid-droplet diameters were quantified from five independent experiments. *G* and *H*, western blots and quantifications for β-catenin and Tcf1 using lysates of WT or Rab8a^−/−^ MEFs before and after induction. β-Actin was used as a loading control. ∗*p* < 0.05; ∗∗*p* < 0.01; ∗∗∗∗*p* < 0.0001 when compared with uninduced conditions. MEFs, mouse embryonic fibroblasts.

To examine whether Rab8 is expressed during MEF differentiation to adipocyte, WT MEFs were first grown to confluency *in vitro* to induce growth arrest (36), followed by treatment with an adipogenic “cocktail” (3-isobutyl-1-methylxanthine, insulin, dexamethasone, and rosiglitazone, a PPAR-γ selective agonist) that stimulates adipocyte differentiation (59, 60, 61). Cells were harvested before induction (uninduced, U), 48 h after adipogenic cocktail stimulation (induced, I) and 10 days after induction (differentiated, D). Western blots detected elevated abundances of Fabp4, Glut4, Rab8, and Rab11 proteins in differentiated cultures compared with uninduced MEFs (Fig. 3*A*). Adipocyte differentiation was confirmed by the formation of lipid droplets detected by BODIPY staining (Fig. 3*B*). Consistent with previous studies (62, 63, 64), Erk1/2 phosphorylation was modulated during this induction and differentiation process (Fig. 3*A*).

Before adipogenic induction, Rab8a^−/−^ MEFs contained small intracellular lipid droplets that were of a similar size to those of WT MEFs (Fig. 3, *C* and *D*). To test if Rab8a deficiency might impact MEFs’ response to adipogenic stimuli, we treated Rab8a^−/−^ MEFs with the same induction cocktail. Fluorescence staining did not detect the formation of large lipid droplets in Rab8a^−/−^ MEFs after treatment with the differentiation protocol (Fig. 3, *E* and *F*). The average diameter of WT droplets increased by over 20-fold upon differentiation, while the droplets in Rab8a^−/−^ MEFs increased by less than 2-fold (Fig. 3, *C*–*F*, data represent five independent experiments).

Western blots showed that induced WT cultures had a decreased abundance of total β-catenin and Tcf1 compared with uninduced WT MEFs, indicating reduced canonical Wnt signaling upon induction (Fig. 3, *G* and *H*, data represent three independent experiments). In contrast, induced Rab8a^−/−^ MEFs exhibited elevated abundances of both total β-catenin and Tcf1 (Fig. 3, *G* and *H*). These data demonstrated that the impaired Rab8^−/−^ MEF differentiation was accompanied by aberrantly elevated Wnt signaling, an observation consistent with the above biochemical analysis (Figs. 1 and 2).

**Figure 4:**
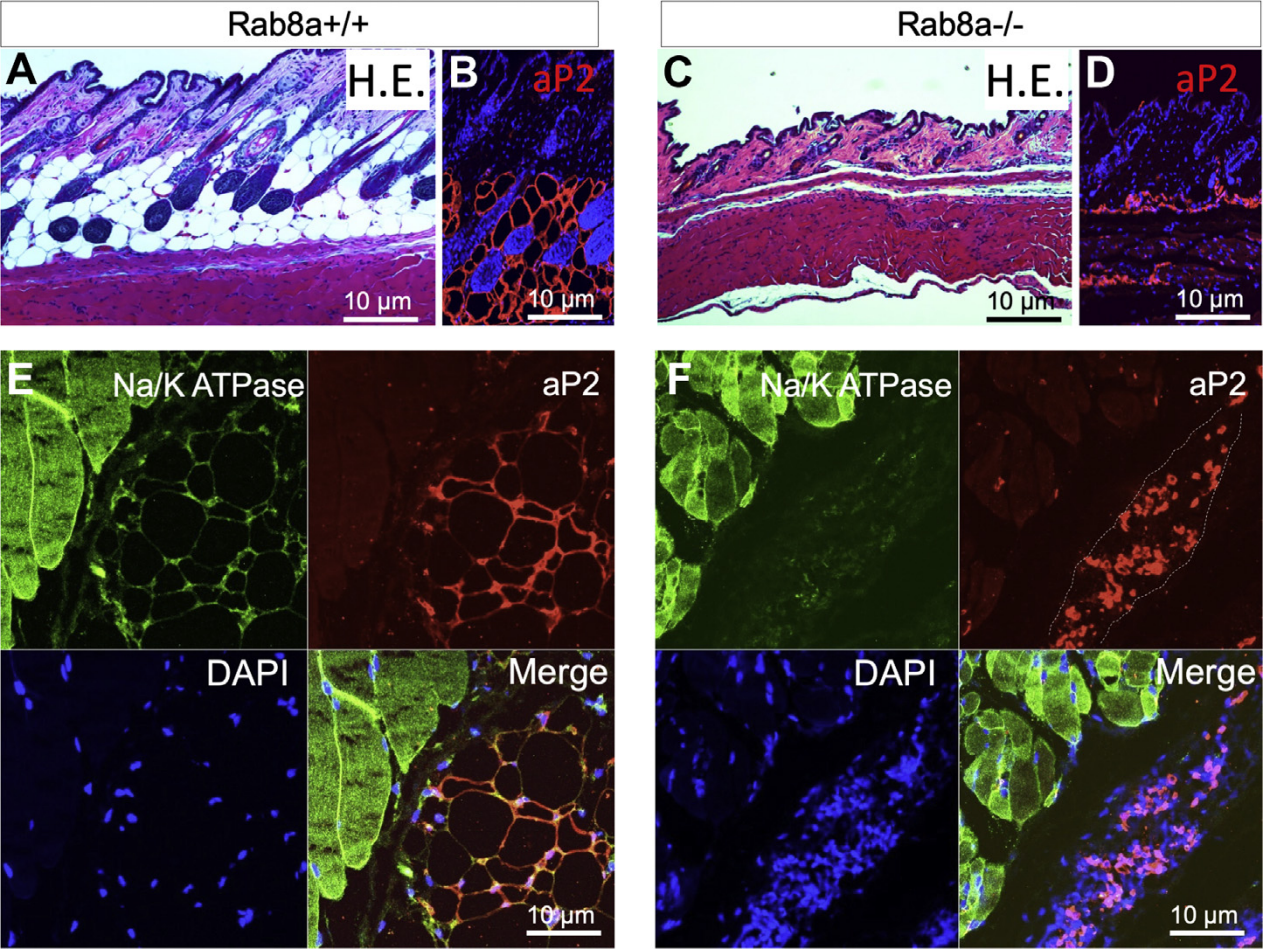
**Rab8a**^**−/−**^**neonates had a reduced number of mature adipocytes.***A* and *C*, H&E staining of the skin with the subcutaneous adipose layer from P0 WT and Rab8a^−/−^ littermates. *B* and *D*, immunofluorescence staining for aP2 (*red*) was performed on matched skin sections of P0 WT and Rab8a^−/−^ littermates. Nuclei was stained by DAPI. *E* and *F*, immunofluorescence staining for aP2 (*red*) using skeletal muscle tissues of P0 WT and Rab8a^−/−^ littermates. Muscle cells were marked by Na/K ATPase (*green*), and nuclei were stained by DAPI (*blue*). *Dotted line circled* aP2-positive cell population in Rab8a^−/−^ tissues. These cells did not demonstrate typical morphology of mature adipocytes shown in the WT tissues.

Examination of the subcutaneous adipose tissues in newborn (P0) Rab8a^−/−^ mice revealed a clearly reduced subcutaneous adipose layer compared with WT littermate pups (Fig. 4, *A* and *C*). Although there was the presence of subcutaneous cells marked by aP2 (red, also known as Fabp4) in Rab8a^−/−^ mice, these cells did not appear to contain the characteristic lipid droplets that were densely packed as the aP2 subcutaneous adipocytes in WT mice (Fig. 4, *B* and *D*). Similar observations for aP2-labeled cells were made in anatomically matched adipocyte tissues adjacent to skeletal muscles (Fig. 4, *E* and *F*). Together, these results suggested that Rab8a is necessary for adipose tissue development *in vivo* and *ex vivo*.

### Loss of Rab8a and Rab8b diminished lipid droplets

**Figure 5:**
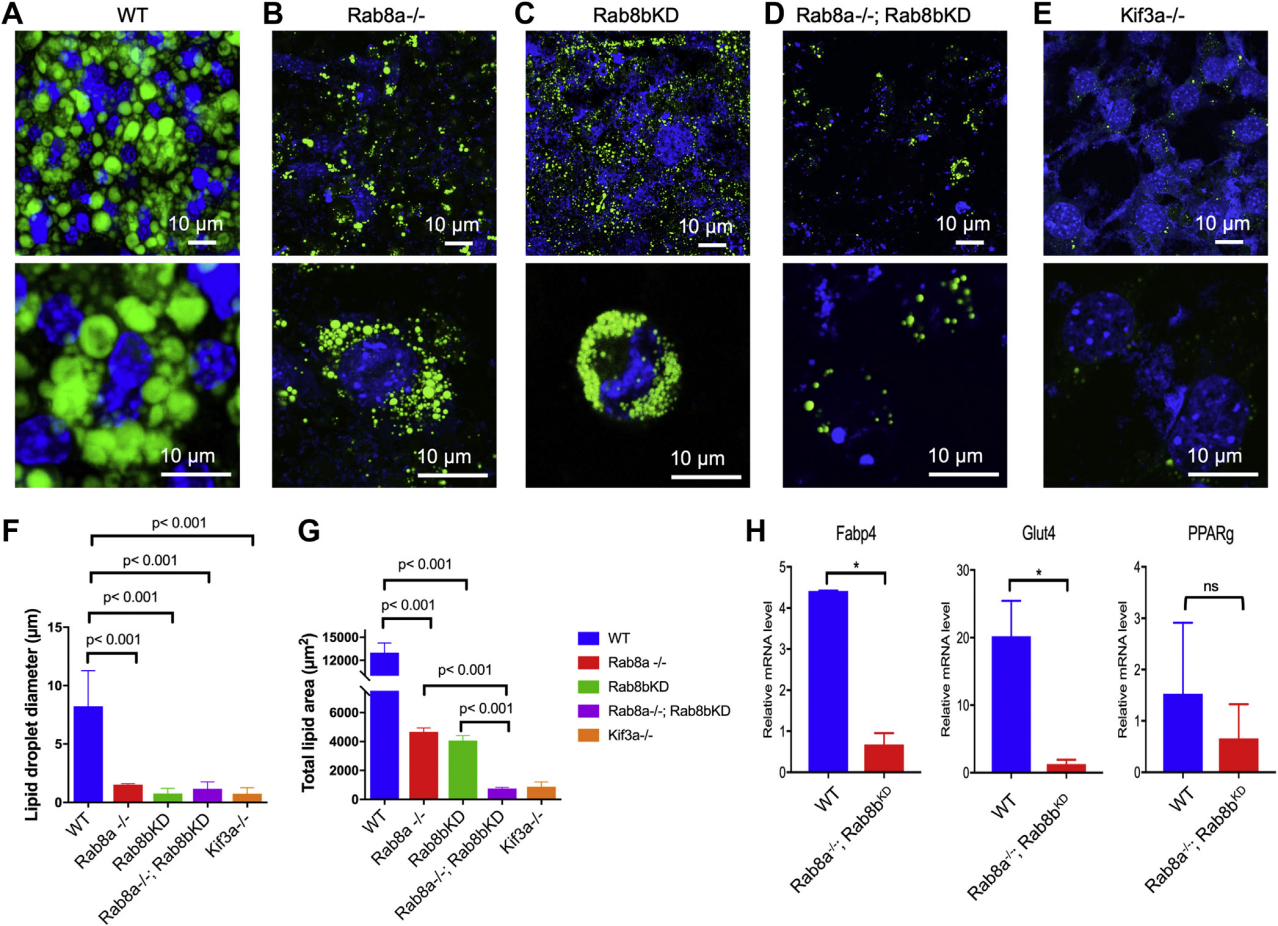
**Combined loss of Rab8a and Rab8b diminishes lipid-droplet formation *ex vivo*.***A*–*E*, representative immunofluorescence micrographs of lipid-droplet staining (BODIPY 493/503; *green*) in WT, Rab8a^−/−^, Rab8b^KD^, Rab8a^−/−^;Rab8b^KD^, or Kif3a^−/−^ MEFs after 2 weeks of induced adipocyte differentiation. Nuclei were stained in *blue*. *F*, quantifications for individual lipid-droplet diameter. *G*, quantifications for the overall areas of lipid droplets in WT, Rab8a^−/−^, Rab8b^KD^, Rab8a^−/−^;Rab8b^KD^, or Kif3a^−/−^ cultures. Data in panels *F* and *G* represent 171, 110, 242, 101, and 101 lipid droplets for each of above genotypes. *p* values were determined by one-way ANOVA. *H*, quantitative real-time RT-PCR of proadipocyte marker transcripts Glut4, Fabp4, and PPAR-γ in differentiated WT or Rab8a^−/−^;Rab8b^KD^ MEFs. *p* values were determined by Student's *t* test. ∗*p* < 0.05, when compared with WT. MEFs, mouse embryonic fibroblasts.

Rab8b^KD^ MEFs exhibited an overall similar Lrp6 profile as Rab8a^−/−^ MEFs. Thus, we next examined whether Rab8b^KD^ MEFs had a similar adipogenic defect. Similar to Rab8a^−/−^ MEFs, the differentiation of Rab8b^KD^ MEFs was also impaired: there was a prolonged delay, taking approximately 13 days, to start the formation of significantly smaller lipid droplets than WT cultures (Fig. 5, *A*–*C*, *F* and *G*).

To further test if there remained some redundancy between Rab8a and Rab8b, we tested adipogenic capacity of Rab8a^−/−^;Rab8b^KD^ MEFs. Rab8a^−/−^;Rab8b^KD^ MEFs, upon induction by the same protocol, failed to attach to glass coverslips around day 5 and 6 of the differentiation protocol. To ameliorate this attachment issue, we coated glass coverslips with 0.1% gelatin. After 2 weeks of induction, Rab8a^−/−^;Rab8b^KD^ MEFs only formed scattered, small lipid droplets, with a significantly decreased total BODIPY-stained lipid area compared with Rab8a^−/−^ or Rab8b^KD^ MEFs (Fig. 5*G*). However, the diameter or the size of lipid droplets in Rab8a^−/−^;Rab8b^KD^ cultures did not differ from those in Rab8a^−/−^ or Rab8b^KD^ MEFs (Fig. 5*F*). Owing to defects in primary cilia, MEFs lacking Kif3a failed to undergo adipogenesis (36), so we used Kif3a^−/−^ MEFs as a reference to assess the degree of adipogenic impairment of Rab8a^−/−^;Rab8b^KD^ MEFs. Quantification of the lipid-droplet size and total area indicated a similar extent of impairment in Rab8a^−/−^;Rab8b^KD^ MEFs as Kif3a^−/−^ MEFs (Fig. 5, *F* and *G*), which also displayed a delayed formation and fewer lipid droplets (Fig. 5*E*). These data suggested a defective activation of the adipogenic program in Rab8-deficient MEFs, which was further supported by significantly reduced transcripts of adipocyte differentiation, including Fabp4 and Glut4 in these Rab8a^−/−^;Rab8b^KD^ MEFs compared with WT MEFs (Fig. 5*H*). PPAR-γ expression was only slightly reduced in Rab8a^−/−^;Rab8b^KD^ MEFs.

### Intact cilia induction and maintenance in the absence of Rab8

Adipogenesis requires the transient induction of the primary cilium in the early stages of differentiation (3), a process requiring Kif3a (16, 36). Our above data suggested that loss of Rab8 shared some common characteristics with the loss of Kif3a (Fig. 5, *E*–*G*), in disrupting MEF differentiation into adipocytes. As Rab8 has been implicated in ciliary cargo transport and cilia development (11, 12, 13, 17, 18), and the primary cilium inhibits Wnt/β-catenin signaling activity (2, 25, 27), we then examined if Rab8-deficient MEFs might exhibit defects in primary cilia development and functionality, none of which has been examined during induced MEF adipogenesis.

**Figure 6:**
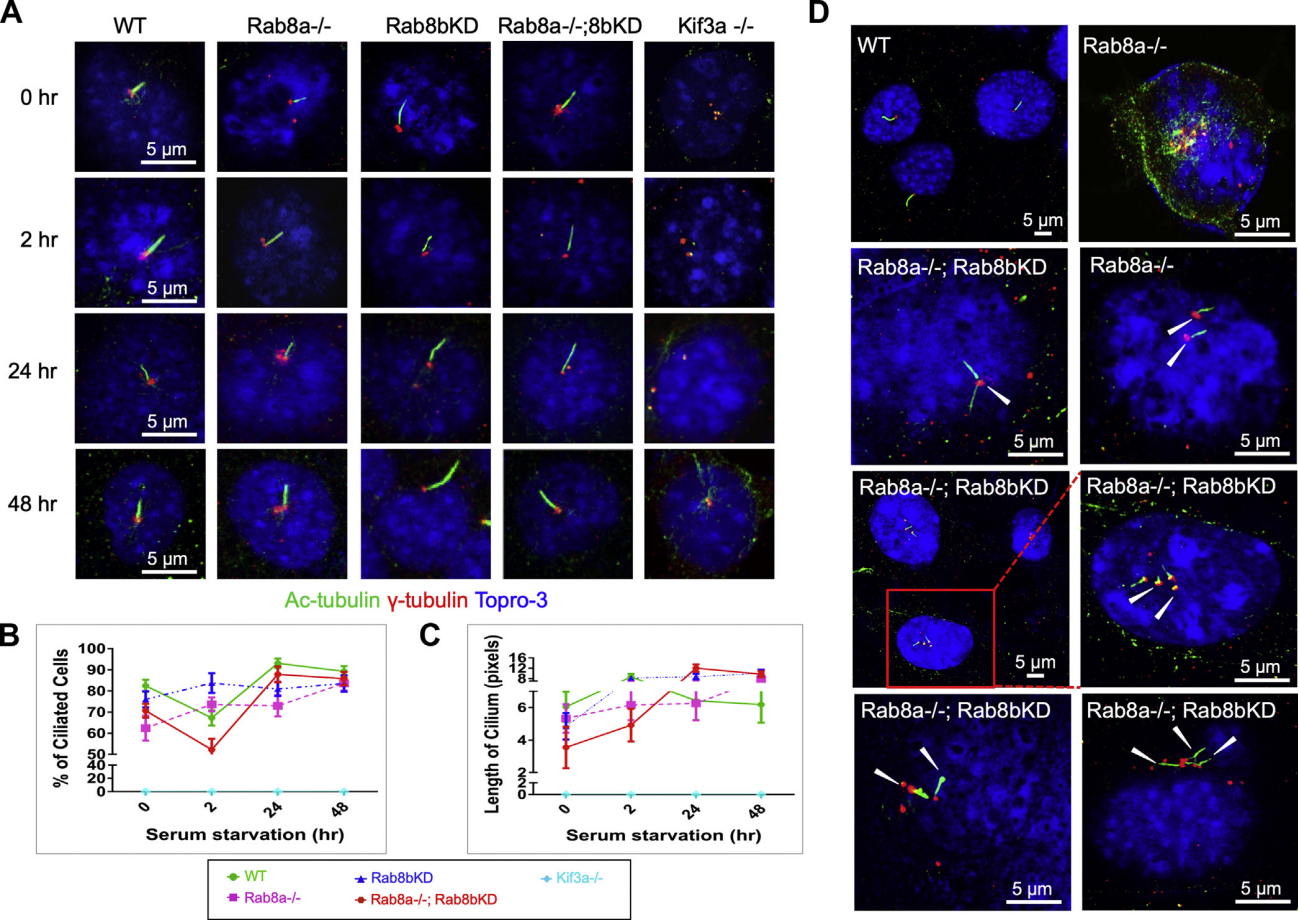
**Intact cilia growth and partial structural defects in Rab8-deficient MEFs.***A*, representative immunofluorescent images of the primary cilia that were labeled by acetylated tubulin (*green*). The basal body was marked by γ-tubulin (*red*) and nuclei by DAPI (*blue*) in WT, Rab8a^−/−^, Rab8b^KD^, or Rab8a^−/−^;Rab8b^KD^, and Kif3a ^−/−−/−^ MEFs. Cilia induction was observed after growth arrest on a time course of 48 h after serum starvation. *B* and *C*, quantification of the percentage of ciliated cells and the average length of a cilium that was measured in pixels. Graphs showed quantifications from six fields of each genotype, containing approximately 20 to 30 cells per time point. Experiments were repeated five times. *D*, representative immunofluorescence of ciliary defects. *White arrowheads* pointed to multiciliated cells, multiple cilia at one basal body, or multiple basal bodies in Rab8a^−/−^ or Rab8a^−/−^;Rab8b^KD^ cells. MEFs, mouse embryonic fibroblasts.

To induce primary cilia formation, MEFs were grown as confluent monolayers for 48 h, serum-starved for 0, 2, 24, or 48 h and then immunofluorescently stained for acetylated tubulin (a cilium marker) and γ-tubulin (a basal body marker) at each time point (Fig. 6*A*). Before serum starvation (0 h), Rab8a^−/−^ MEFs showed the lowest percentage (62%) of ciliated cells, followed by Rab8a^−/−^;Rab8b^KD^ MEFs (71%), Rab8b^KD^ MEFs (76%), and WT MEFs (82%). As expected, primary cilia were not observed in Kif3a^−/−^ MEFs (Fig. 6, *A* and *B*).

Rab8a^−/−^;Rab8b^KD^ MEFs exhibited the shortest primary cilia before serum starvation, followed by Rab8a^−/−^ or Rab8b^KD^ MEFs, whereas WT MEFs possessed the longest primary cilia (Fig. 6*C*). After serum starvation, the percentage of ciliated cells and the cilium length increased in WT and the three Rab8-deficient MEF cell lines (Fig. 6*C*). After 24 h of serum starvation, 92.8% of WT MEFs, 76% of Rab8a^−/−^ MEFs, 85.4% of Rab8b^KD^ MEFs, and 88.0% of Rab8a^−/−^;Rab8b^KD^ MEFs were ciliated. After prolonged serum starvation (48 h), all MEF cell lines retained primary cilia (90.5% for WT; 83.7% for Rab8a^−/−^;84.9% for Rab8b^KD^, and 85.6% for Rab8a^−/−^;Rab8b^KD^). Kif3a^−/−^ MEFs remained nonciliated at all time points examined (Fig. 6, *A* and *B*).

Although the absence of either single or double Rab8 did not appear to prevent the formation of the primary cilium, a fraction of Rab8a^−/−^ and Rab8a^−/−^;Rab8b^KD^ MEFs contained multiple basal bodies and aberrant cilia (Fig. 6*D*, inset). Interestingly, in some Rab8a^−/−^;Rab8b^KD^ MEFs, multiple cilia grew from a single basal body, while some other Rab8a^−/−^;Rab8b^KD^ cells contained two sets of basal body and cilia (Fig. 6*D*, white arrowheads). These changes were rarely observed in WT or Rab8b^KD^ MEFs. Thus, although the overall growth of a primary cilium may not require Rab8, the loss of both Rab8a and Rab8b did appear to affect basal body duplication and the morphological outcome of primary ciliogenesis.

### Rab8-dependent Fzd2 traffic to primary cilia

**Figure 7:**
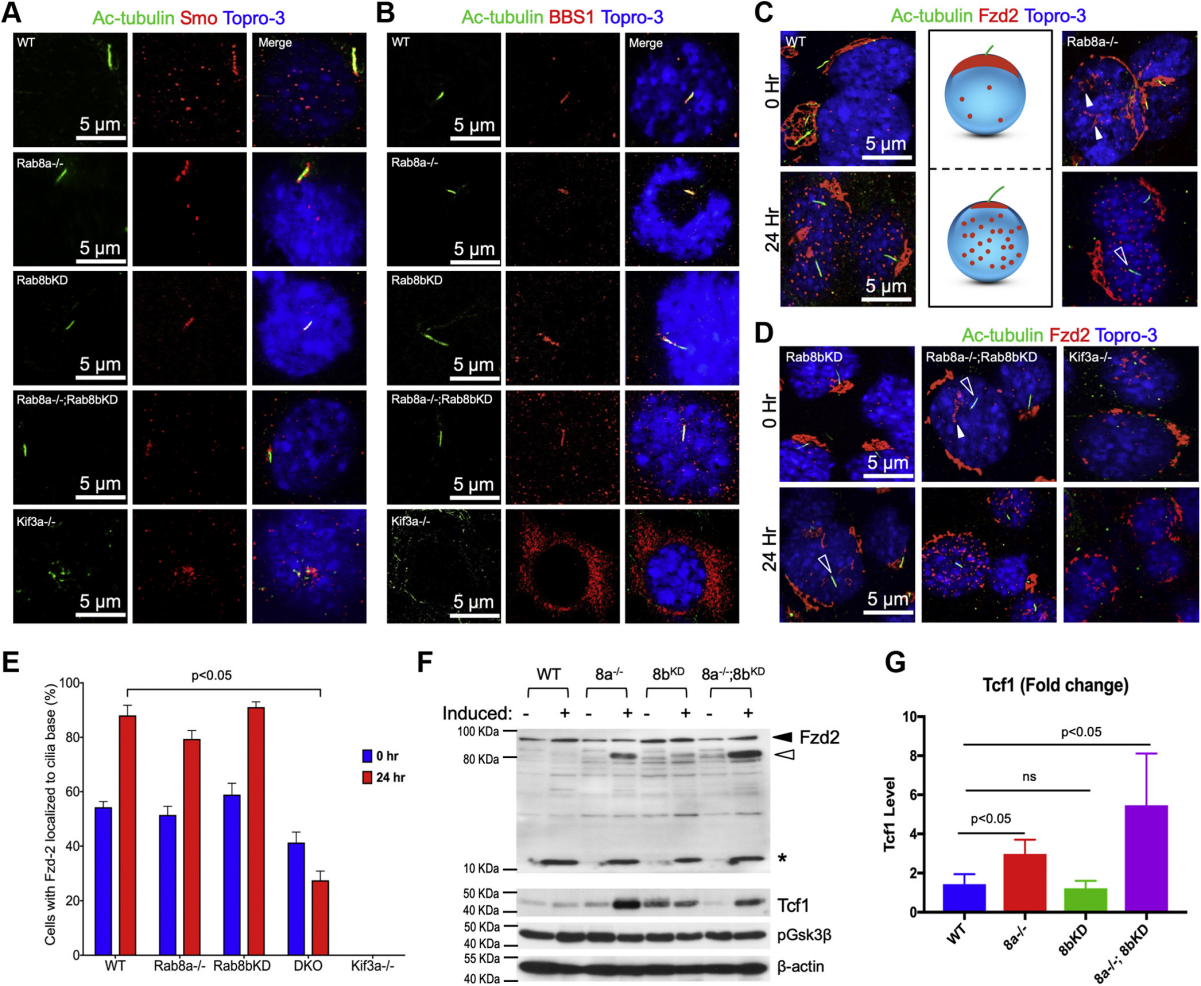
**Aberrant Fzd2 translocation and processing in Rab8-deficient MEFs during adipogenic induction.***A* and *B*, representative immunofluorescent images of Smo or BBS1 that were localized at the primary cilia (acetylated tubulin; *green*), in WT, Rab8a^−/−^, Rab8b^KD^, Rab8a^−/−^;Rab8b^KD^, or Kif3a^−/−^ MEFs. *C* and *D*, representative immunofluorescent images of Fzd2 (*red*) in MEFs before or 24 h after cilia induction. A diagram is used to summarize the findings in WT cells: Fzd2 appeared as a cilium-associated patch before induction; after induction, numerous Fzd2 puncta or vesicles appeared (*red dots*). *White arrowheads* point to increased Fzd2 puncta that were not associated with a cilium in Rab8a^−/−^ and Rab8a^−/−^;Rab8b^KD^ MEFs before induction. *Open white arrowheads* point to cilia that were not associated with a patch of Fzd2. *E*, quantification of the percentage of cells with Fzd2 localized to the base of the primary cilium at 0 (*blue bar*) or 24 h (*red bar*) after cilia induction. *F*, western blots for Fzd2, Tcf1, and β-catenin were performed on total lysates of WT or Rab8-deficient MEFs under uninduced or induced conditions. Full-length Fzd2 was marked by a *solid black arrowhead*; truncated Fzd2 marked by an *empty arrowhead*; cleaved fragment marked by an *asterisk*. *G*, fold changes in Tcf1 protein abundance between uninduced and induced MEFs were quantified from three independent experiments. *p* values were determined by *t* test. MEF, mouse embryonic fibroblast.

Although Rab8 deficiency did not prevent the formation of the primary cilium in our analysis, literature suggested that there might be abnormal transport of certain ciliary cargos in the absence of Rab8 (17). We first examined Smo, a 7-pass transmembrane protein trafficked to the primary cilium to modulate Hh signaling activity, and it is moved along the cilium by IFT (65, 66). Smo was properly localized to the primary cilia in WT, as well as Rab8a^−/−^, Rab8b^KD^, and Rab8a^−/−^;Rab8b^KD^ MEFs (Fig. 7*A*). Likewise, BBS1, a subunit of the Bardet–Biedl Syndrome protein complex shown to interact with Rab8, was also localized to primary cilia in WT and Rab8-deficient MEFs (Fig. 7*B*). Thus, ciliary transport of Smo and BBS1 was not affected by Rab8 deficiency in MEFs.

Planar cell polarity effectors are trafficked to the base of primary cilia through a mechanism dependent upon Fuzzy recruitment of Dishevelled to Rab8 vesicles (27, 29, 67). The planar cell polarity activity directly intersects with Wnt signaling (68). As we have shown that Rab8-deficient MEFs had enhanced Wnt signalosome activities, we immunofluorescently stained Fzd2, a Wnt receptor, in cilium-induced WT and Rab8-deficient MEFs. In 55% WT MEFs, Fzd2 was localized to large membrane “patches” at the base of the primary cilium before serum starvation (0 h, Fig. 7, *C* and *E*). After 24 h of starvation, patches of Fzd2, at the base of primary cilia, was detected in 88.7% of WT cells and numerous small Fzd2-stained puncta (Fig. 7, *C* and *E*). Approximately 50% of Rab8a^−/−^ MEFs also displayed Fzd2 membrane patches localized to primary cilia and that was increased to 80% after 24 h of serum starvation. In addition, Rab8a^−/−^ MEFs contained more fluorescent Fzd2 puncta even before serum starvation, and these puncta increased after 24 h of serum starvation (arrowheads, Fig. 7, *C* and *E*). Although Rab8a^−/−^ and Rab8b^KD^ MEFs had approximately equal numbers of Fzd2 patches at the base of cilia, in Rab8b^KD^ MEFs, the cilium-associated patches appeared relatively small and Rab8b^KD^ cells lacked puncta either before or after starvation (Fig. 7*C*). Rab8a^−/−^;Rab8b^KD^ MEFs were found to have fewer cilium-associated Fzd2 patches before starvation, and this deficiency became significant after 24 h of starvation (Fig. 7, *D* and *E*). The formation of Fzd2 puncta in Rab8a^−/−^;Rab8b^KD^ cells was not impaired (Fig. 7*D*). Kif3a^−/−^ MEFs, while lacking primary cilia, still contained Fzd2 membrane patches and puncta (Fig. 7*D*).

The above data indicated abnormal Fzd2 trafficking in the absence of Rab8 during adipogenic induction. We examined Fzd2 protein in various MEFs by Western blots before and after adipogenic induction. Full-length Fzd2 appeared in all cell lines before and after adipogenic induction (solid arrowhead, Fig. 7*F*). Interestingly, we observed the induction of a truncated Fzd2 that was prominent in Rab8a^−/−^ and Rab8a^−/−^;Rab8b^KD^ MEFs, and to a lesser extent in Rab8b^KD^ (empty arrowhead, Fig. 7*F*), suggesting that lack of Rab8 led to a possible post-translationally modified Fzd2. Rab8a^−/−^ and Rab8a^−/−^;Rab8b^KD^ MEFs also showed a concomitant induction of Tcf1 that was not seen in WT or Rab8b^KD^ cells (Fig. 7, *F* and *G*, data represent three independent experiments). Together, these data collectively suggested that Rab8 plays a critical role in regulating the membrane positioning of Wnt signaling components and proper MEF differentiation in response to adipogenic signals.

## Discussion

We used genetic and biochemical approaches to demonstrate the impact of Rab8 deficiency on the morphogenesis, differentiation, and signaling of mouse embryonic fibroblasts. Our data suggested a Rab8-dependent membrane traffic of signaling receptors and intracellular positioning of signalosomes in Wnt-receiving MEFs. As attenuation of Wnt signaling is essential for adipogenesis (22, 69), the aberrant activation of this pathway in Rab8-deficient MEFs is likely responsible for the severe impairment of MEF differentiation into adipocytes.

Rab8a-deficient MEFs were hypersensitive to Wnt ligand stimulation. In serum-starved Rab8a^−/−^ MEFs, there were higher basal pLrp6, the core unit of the Lrp6 signalosome, at the plasma membrane and intracellularly. As pLrp6 assembles downstream signaling components, the increased positioning of Lrp6 vesicular machinery at the plasma membrane and in the cytoplasm could be poised to induce a robust signaling output upon ligand stimulation. This may explain the enhanced ligand sensitivity and signaling in Rab8a^−/−^ MEFs. Active Rab8-driven anterograde membrane trafficking may help diminish Lrp6 intracellular aggregation. Thus, we propose that in Wnt-receiving MEFs, normal Rab8 membrane trafficking may allow the clearance of the Lrp6 signalosome when there is minimal extracellular ligand, thereby preventing unwanted activation of Wnt signaling. Such Rab8 trafficking appears to be particularly important for Wnt signaling attenuation during adipogenic induction and for MEFs to differentiate into adipocytes. This is conceptually important as this Rab8-dependent mechanism may offer additional insight into how Wnt-β-catenin signaling is controlled in differentiating cells.

Our work adds to the existing literature that documented the regulation of Wnt-β-catenin signaling by Rab11, Rab14, Rab25, and Rab27 (70, 71, 72, 73). In terms of regulating adipogenesis of MEFs, Rab8a and Rab8b exhibit similar contributions. A redundancy between the two factors was revealed in Rab8a^−/−^;Rab8b^KD^ MEFs, which showed a dramatic reduction of the formation of lipid droplets even after a prolonged differentiation phase. However, Rab8a and Rab8b do display differences with regard to cilia development and Fzd2 transport. In these scenarios, lack of Rab8a induced a stronger phenotype than the lack of Rab8b. In zebrafish, Rab8b contributes to Lrp6 endocytosis and Wnt signaling (74). Exogenous Wnt stimulation is known to induce Lrp6 internalization to lipid raft domains to sequester Axin and prevent β-catenin degradation (75, 76). Internalized Lrp6 vesicles have been colocalized with Rab7 endosomes and mature into multivesicular bodies to sequester GSK3-β to sustain Wnt-β-catenin signaling (77). Our sedimentation analysis showed an impact of Rab8 deficiency on endocytic compartmentalization, suggesting that Rab8 vesicular traffic is essential to maintain homeostasis in the network of endocytosis and exocytosis. This homeostasis must be critical for the activation and inhibition of Wnt-β-catenin signaling.

Mesenchymal stem cells are pluripotent cells capable of differentiating into various cell fates including the fat, bone, and cartilage. Wnt-β-catenin signaling inhibits adipogenesis, and several Wnt ligands such as Wnt3, Wnt6, Wnt10a, and Wnt10b have been identified as adipogenic inhibitors (1, 3, 22, 23). It should also be noted that Wnt3a induced *de novo* lipid-droplet formation in L cells and hepatocytes (78). In undifferentiated mesenchymal stem cells, Wnt-β-catenin signaling was shown to inhibit the expression of proadipogenic factors such as C/EBPs and PPAR-γ (79). When these cells are induced to differentiate, C/EBP-α, C/EBP-β, and PPAR-γ expression levels increase and inhibit nuclear translocation of β-catenin; the inhibition of nuclear localization of β-catenin allows cells to initiate an adipogenic transcriptional program (79). Suppression of PPAR-γ signaling by noncanonical Wnt signaling and tumor necrosis factor-alpha/Interleukin-1 signaling steers cells toward an osteogenic fate by suppressing adipogenesis, thus highlighting how tight regulation of these cell-fate determinants maintains tissue homeostasis (1, 3, 4, 6, 22, 36). These studies are largely consistent with our observation that an enhanced Wnt signaling in Rab8-deficient MEFs impaired adipogenic potential. We also found that Rab8 abundance is increased in mature adipocytes (8) and we speculate it may function to attenuate Wnt signaling, to allow adipocyte differentiation and maturation.

We also observe an interesting localization of Fzd2 to a disk of membrane at the base of the primary cilium. In *Drosophila*, Fzd2 has been implicated in a nuclear import pathway during synaptic development in neuromuscular joints. In that model, Fzd2 is endocytosed and translocated to the perinuclear area where it is cleaved (80, 81, 82). The C-terminus-containing fragment of Fzd2 is then translocated to the nucleus *via* importin-β11, importin-α2, and GRIP (a PDZ protein) (81, 82). It was also proposed that nuclear Fzd2 may act as a transcription factor to regulate synaptic development (82). We observed that in MEFs, there was a redistribution of Fzd2 upon adipogenic induction, and this redistribution appeared to correlate with a prominent processing (possibly a cleavage event) of this receptor into a smaller fragment. A distinct processing product was detected prominently in Rab8a^−/−^ and Rab8a^−/−^;Rab8b^KD^ MEFs upon induction, suggesting that loss of Rab8a affected Fzd2 processing. We currently do not know the significance of this change, but the aberrant Fzd2 processing in the two above-mentioned MEF lines appeared to correlate with Tcf1 induction and, thereby, Wnt pathway activity. Collectively, Rab8 appears to be critical in the maintenance, distribution, and compartmentalization of key Wnt pathway molecules (*i.e.*, Fzd and LRP6). Future studies will elucidate the precise molecular mechanisms of such regulation.

Transient formation and disassembly of primary cilium during adipogenesis (3, 5, 24) may be correlated with Wnt signaling and β-catenin degradation (2, 25, 27). Ciliary proteins are known interacting partners of Rab8. For example, the BBSome directly interacts with Rab8 at the basal body during primary cilium formation and elongation (17). Polycystin-1, a ciliary targeting signal that regulates cell polarization, also complexes with Rab8, and its trafficking is dependent on Rab8 and Arf6 (35). IFT particles are scaffolded with Rab8 through a protein called Elipsa during trafficking events within the cilium itself (21). Rab8b interacts with otoferlin, a crucial ciliary protein in cochlea; loss of otoferlin leads to hearing impairment in patients. The otoferlin and Rab8b complex is speculated to regulate tethering and fusion of endosomes (32). While depletion of either Rab8a or Rab8b was shown to be sufficient to inhibit ciliogenesis in some studies (13, 14, 18, 35), we found that Rab8a- and Rab8b-deficient MEFs formed intact cilia (58). Rab8-deficient MEFs only showed some ciliary morphology defects, namely fewer ciliated cells, slightly shorter length, and multiciliated cells. In addition, the formation of the primary cilia was not grossly attenuated in cells depleted for both Rab8a and Rab8b. Nevertheless, Rab8a^−/−^;Rab8b^KD^ MEFs exhibited the strongest defect in adipogenesis, to an extent similar to Kif3a^−/−^cells that had both impaired ciliogenesis and adipogenic potential (16). We postulate that the assembly of signaling components by Rab8 at the plasma membrane, and not the formation of a primary cilium, may thus be a critical determinant of adipogenesis. The molecular control of adipocyte maturation by Rab proteins may be exploited in the future to better understand how fat tissue is dysregulated in disease and to guide the design of effective therapeutic strategies.

## Experimental procedures

### Mice, MEF cell isolation, and culture

Animal studies were approved by the Institutional Animal Care and Use Committee of Rutgers University. Mice carrying the Rab8a null allele have been described previously (15, 41). Rab8a+/− mice were set up for plug mating. On day E12.5, the pregnant female was sacrificed, and the embryos were dissected out. After removing the placenta, yolk sac, and uterus, the head and internal organs (the heart, liver, spleen, gut) were removed. Tails were saved for genotyping. The remaining tissue was placed into 1.5-ml Eppendorf tubes with trypsin-EDTA. With sterile scissors, the tissue was diced as finely as possible and incubated in a 37 °C humidified chamber for 10 min. After suspension, cells were pelleted and plated in Dulbecco's modified Eagle's medium (DMEM) containing sodium pyruvate, 10% fetal bovine serum (FBS), 1.0 mg/ml Pen-Strep, and 0.05 mg/ml gentamicin. Cells were passaged three times to isolate fibroblasts before starting experiments.

### Cloning and lentiviral KD of Rab8b

Rab8b-specific lentiviral shRNA construct targeting against the 3'UTR of mouse Rab8b was constructed by inserting the annealed complementary oligonucleotides (5'-CCGGGCCAAGAACTAACAGAACTTTCCATGGAAAGTTCTGTTAGTTCTTGGCTTTTTG-3' and 5'-AATTCAAAAAGCCAAGAACTAACAGAACTTTCCATGGAAAGTTCTGTTAGTTCTTGGC-3') into pLK0.1 lentiviral vector (Addgene) between AgeI and EcoRI sites. For viral packaging, this lentiviral vector along with pVSV-G was transfected into GP2-293 cells (Clontech) using Lipofectamine 2000 (Invitrogen). After 48 h, the supernatant was collected and subjected to ultracentrifugation (15,000*g*, 2 h) for viral concentration. The viral pellet was resuspended with 200 μl of DMEM and aliquoted for later usage. For Rab8b KD, MEFs were infected with diluted lentivirus stock (1:50,000) for 5 h in DMEM in the presence of polybrene (8 μg/ml) and then incubated with complete DMEM containing 10% FBS for another 24 h. Puromycin (3 μg/ml) was added into the culture medium for selection. KD efficiency and the maintenance of KD were confirmed by Western blotting.

### Cell culture and adipocyte induction

Cells were continuously maintained in 5% CO_2_, 37 °C humidified chamber. MEF cells were maintained at 70% confluency in DMEM containing sodium pyruvate, 10% FBS, 1.0 mg/ml Pen-Strep, and 0.05 mg/ml gentamicin. Adipogenesis of MEF cells was performed according to previously published protocols (59, 60, 61). To induce adipogenesis, cells were grown to 100% confluency and then maintained as an overcrowded plate for 48 h. Cells were incubated in DMEM containing sodium pyruvate, 10% FBS, 1.0 mg/ml Pen-Strep, 0.05 mg/ml gentamicin, 1.0-μM dexamethasone, 0.5-mM 3-isobutyl-1-methylxanthine, 5.0 mg/ml insulin, and 1.0-μM rosiglitazone for 48 h. Cells were then incubated in DMEM containing sodium pyruvate, 10% FBS, 1.0 mg/ml Pen-Strep, 0.05 mg/ml gentamicin, and 5.0 mg/ml insulin for 8 days. Full differentiation was achieved within 12 days from induction. Lipid droplets of adipocytes were stained by BODIPY 493/503 and quantified by ImageJ. For staining experiments, cells were plated and/or differentiated on 0.1% gelatin-coated coverslips in 6-well plates. For cell lysates, cells were plated and differentiated for 12 days in 10-cm^2^ culture plates before harvesting.

### Luciferase assay

MEF cells were plated in 24-well plates and transfected in duplicate with 250-ng TopFlash, 10-ng Renilla, and 200-ng β-catenin ΔN using Lipofectamine 3000 (Invitrogen). 24 h after transfection, 100% confluent cells were serum-starved for 8 h and treated with 50 ng/ml Wnt3a (315-20; PeproTech) dissolved in PBS or 20-μM CHIR99021 (Stemgent) for 16 h. For β-catenin ΔN group, 100% confluent cells were serum-starved for 24 h. Cells were then lysed, and the luciferase assay was carried out by using the Dual-Luciferase Reporter Assay Kit (Promega) and the GloMax-Multi Detection System (Promega) according to the manufacturer's instruction. pcDNA3 ΔN47 β-catenin was from Addgene (57) (Addgene plasmid # 19287).

### Western blot

After starvation for 16 to 24 h, WT and Rab8a −/− MEFs cells were treated with vehicle or Wnt3a (100 ng/ml) and harvested at 0, 2, and 4 h. MEF cells were washed with ice-cold PBS and lysed in the lysis buffer (50-mM Tris, pH 7.5, 150-mM NaCl, 10-mM EDTA, 0.02% NaN_3_, 50-mM NaF, 1-mM Na_3_VO_4_, 1.0% NP-40, 0.1% SDS, 1-mM PMSF, 0.5-mM DTT, and protease inhibitors) at 4 °C. The lysates were incubated on ice for 15 min and spun down in 4 °C, 17,000*g* for 15 min. The supernatant was collected and analyzed for the protein concentration. After adding lithium dodecyl sulfate and 50-mM DTT, the samples were incubated at 70 °C for 10 min. Samples were loaded on SDS-PAGE gels, transferred to polyvinylidene difluoride membranes, and blocked in 5% skim milk in Tris-buffered saline with 0.1% Tween 20 for 1 h at room temperature (RT). The membrane was incubated with primary antibodies: LRP6 (3395, Cell Signaling Technology), p-LRP6 (S1490) (2568, Cell Signaling Technology), Axin1 (2087, Cell Signaling Technology), Dvl2 (3224, Cell Signaling Technology), Dvl3 (3218, Cell Signaling Technology), β-catenin (1:2000; #610154, BD Transduction Laboratories), p-β-catenin (9561, Cell Signaling Technology), GSK3β (#9315, Cell Signaling Technology), p-GSK3β (9323, Cell Signaling Technology), β-actin (1:2000; sc-47778, Santa Cruz Biotechnology), TCF-1 (2203, Cell Signaling Technology), Caveolin-1 (3267, Cell Signaling Technology), Rab5 (3547, Cell Signaling Technology), Rab7 (9367, Cell Signaling Technology), Rab9 (5118, Cell Signaling Technology), Rab8 (#610844, BD Transduction Laboratories), Rab11a (#R0009/YL8, US Biological), Fzd2 (52565, Abcam), Fabp4 (13979, Abcam), Glut4 (2213, Cell Signaling Technology), Erk1/2 (9102, Cell Signaling Technology), and pErk1/2 (9106, Cell signaling Technology) at 4 °C overnight in a dilution of 1:1000 unless indicated. After washing with 1x Tris-buffered saline with 0.1% Tween 20, the membranes were incubated in the secondary antibody (1:2000; ECL, GE Amersham ECL) for 1 to 2 h at RT and developed in ECL detection reagents (RPN2209 and RPN2232, GE Amersham).

### Sucrose gradient sedimentation

This method is according to the previous study (40). Briefly, after starvation for 16 to 24 h, WT Rab8a^−/−^ and Rab8b^kd^ MEF cells were treated with Wnt3a (100 ng/ml) for 4 h before harvest. Cells were harvested in Hank’s Balanced Salt Buffer on ice, pelleted, and lysed for 20 min in an extraction buffer which contains 30-mM Tris (pH 7.4), 140-mM sodium chloride, 1% Triton X-100, 25-mM sodium fluoride, 3-mM sodium orthovanadate, 2-mM PMSF, and protease inhibitor cocktail tablet (Roche). The lysates were centrifuged, and the supernatant was layered on top of a 15 to 40% sucrose gradient 30-mM Tris (pH 7.4), 140-mM sodium chloride, 0.02% Triton X-100, 25-mM sodium fluoride, 3-mM sodium orthovanadate, and protease inhibitors. Ultracentrifugation was performed in a Beckman SW55Ti rotor at 45,200 rpm for 4 h at 4 °C. After centrifugation, fractions were collected from the bottom of the tube by a peristaltic pump and analyzed by SDS-PAGE and immunoblot.

### Immunofluorescence

For BODIPY 493/503 staining, the powder was dissolved in 100% ethanol to a final concentration of 1 mg/ml. In the dark, the differentiated cells plated on coverslips were fixed in 4% PFA for 10 min, washed in 1x high-quality PBS twice, and incubated in 0.1% Triton X-100 for 10 min at RT. Cells were then incubated in BODIPY 493/503 (1:1000 of stock in 1x PBS) for 30 min at RT and counterstained with TO-PRO-3 (1:500 in 1x PBS) for 15 min at RT. Coverslips were dried and mounted onto slides using the ProLong Anti-fade reagent (Thermo Fisher Scientific) and sealed with clear nail polish. Imaging was performed using a Zeiss LSM 510 Confocal Microscope.

For ciliogenesis, cells were plated in 6-well plates containing coverslips. All cells were grown and maintained in overconfluency for 48 h and then incubated in a serum-free medium for 0, 2, 24, and 48 h, respectively. On the day of staining, cells were fixed in methanol at −20 °C for 5 min. Permeabilization occurred in 0.1% Triton X-100 in PBS for 10 min at RT. Cells were blocked in 10% goat serum in PBS for 30 min at RT and then incubated with primary antibodies: acetylated α-tubulin (1:800; T7451, Sigma-Aldrich), γ-tubulin (1:800; 84355, Abcam) in 10% goat serum in PBS, at 4 °C overnight. On the second day after washing with PBS, cells were incubated with the fluorescently conjugated secondary antibody (1:1000; Thermo Fisher Scientific) in the dark for 1 h at RT. After washing in PBS, cells were counterstained with TO-PRO-3 (1:500; T3605, Thermo Fisher Scientific) in PBS for 15 min at RT. Coverslips were dried and mounted onto slides with the ProLong Anti-fade reagent (P36930, Thermo Fisher Scientific) and sealed with clear nail polish. Imaging was performed using the Zeiss LSM 510 confocal microscope.

Immunostaining was performed as described previously (41, 43, 45). Cells were fixed in 4% paraformaldehyde, blocked in 2% bovine serum albumin, 2% goat serum, and 0.1% Triton X-100 blocking buffer for 1 h, and incubated with primary antibodies, acetylated α-tubulin (1:500; T7451, Sigma-Aldrich) and γ-tubulin (1:500; #ab84355, Abcam), in the blocking buffer overnight at 4 °C. On the following day after washing with PBS, sections were incubated with a fluorescently conjugated secondary antibody (1:1000; Thermo Fisher Scientific) in a blocking buffer for 1 h at RT. Sections were counterstained for nuclei with TO-PRO-3 (1:500; T3605, Thermo Fisher Scientific) in PBS for 15 min at RT and then washed and mounted with the ProLong Gold Anti-fade mounting media (P36930, Thermo Fisher Scientific). Images were taken using a Zeiss LSM 510 confocal microscope.

For LPR6 immunofluorescence detection, cells were transiently transfected with pCS2+LRP6 GFP (83). At 100% confluency, cells were serum-starved for 24 h, followed by treatment with Wnt3a (100 ng/ml, diluted in DMEM) or vehicle only. After 15 min of treatment, cells were fixed in 4% PFA and indirect immunofluorescent straining was subsequently performed as described previously for GFP (1:100; 8334, Santa Cruz Biotechnology) in 4 °C overnight. Images were taken using a Zeiss LSM 510 confocal microscope. The numbers of LRP puncta, or peripheral puncta (based on phalloidin staining), were manually counted from individual cells of 6 to 8 different fields. Data represent a minimum of five independent experiments.

### RNA extraction and qPCR analysis

Uninduced MEFs were harvested at 90% confluency. Induced MEFs were harvested 48 h after the start of induction. RNA extraction was performed by using the RNeasy kit (QIAGEN) according to the manufacturer's instruction. Quantitative PCR analysis was described previously (45, 84). Primer sequences are shown below: Glut4, 5’-CAGATCGGCTCTGACGATG-3’ and 5’-ACTGAAGGGAGCCAAGCAC-3’; Fabp4, 5’-CGCAGACGACAGGAAGGT-3’ and 5’-CAGCTTGTCACCATCTCGTT-3’; PPAR-γ, 5’-AGCCTGTGAGACCAACAGC-3’ and 5’-TGGTTCACCGCTTCTTTCA-3’; β-actin, 5’-TTGCTGACAGGATGCAGAAG-3’ and 5’-CCACCGATCCACACAGAGTA-3’.

### Quantification and statistics

All results represent three or more independent experiments unless stated otherwise. Western blots, dimension of lipid droplets, and the cilia length were measured by ImageJ (NIH, version 1.49). For each cell line, ciliated cells were counted manually against total cells from six low-magnification confocal images. The lipid-droplet number and size were quantified using particle analysis in ImageJ. Data represented the means ± SEM from independent experiments. Statistical analysis was performed by Student’s *t* test, one-way ANOVA, or two-way ANOVA. Significance was accepted at *p* < 0.05. Calculations and graphs were generated using GraphPad Prism (7.04).

## Data availability

All data are contained within the article.

## Supporting information

This article contains supporting information.

## Conflict of interest

The authors declare that they have no conflicts of interest with the contents of this article.
