# Supplementary material for: Rab8 attenuates Wnt signaling and is required for mesenchymal differentiation into adipocytes
Source: J Biol Chem. 2021 Mar 1;296:100488. doi: 10.1016/j.jbc.2021.100488 (PMC8042397; doi:10.1016/j.jbc.2021.100488)
Supplement: Supplemental Figure S1 [file mmc1.pdf]

Rab8 attenuates Wnt signaling and is required for mesenchymal differentiation into adipocytes

Stypulkowski et al.

Supporting Data File:

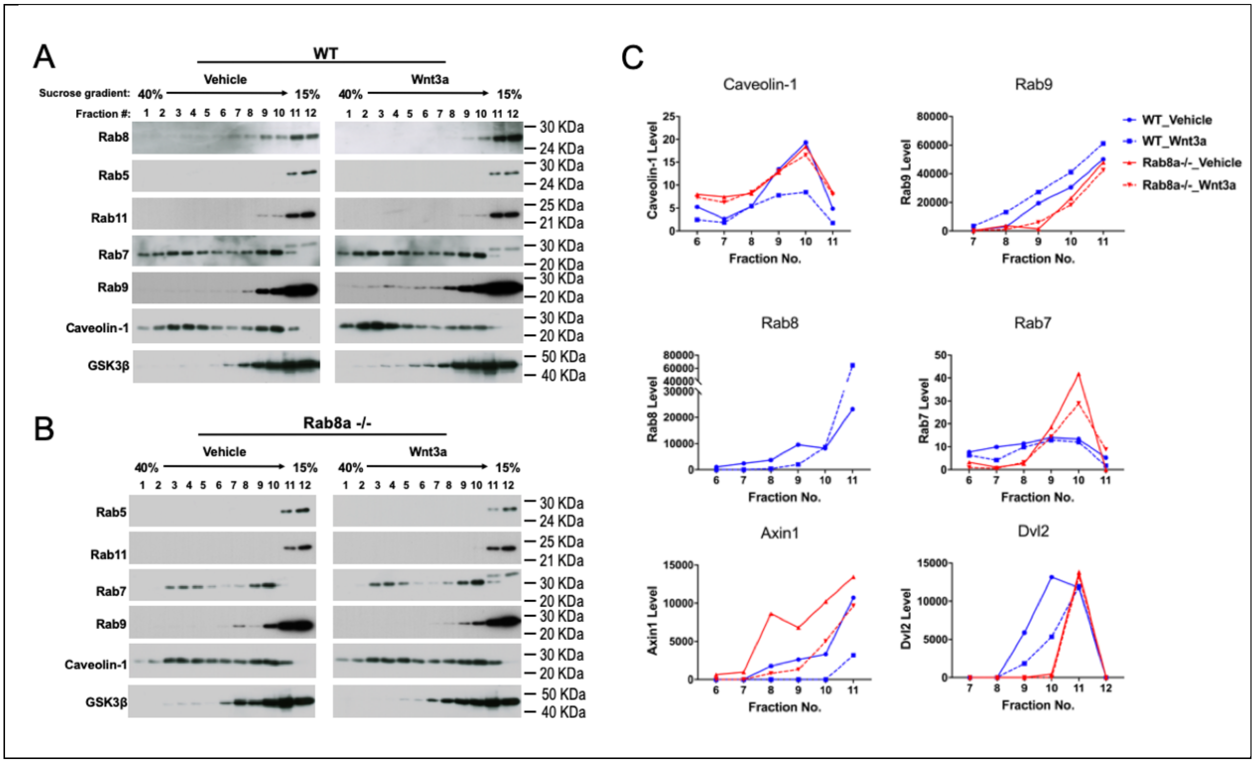

Supplementary Figure 1. Loss of Rab8a alters endocytic and vesicular compartmentalization in MEFs. (A-B) Western blots were performed for Rab8, Rab5, Rab11, Rab7, Rab9, Caveolin-1, Gsk3beta on fractions obtained from sucrose sedimentation of serum-starved WT and Rab8a-/- MEFs treated with vehicle or Wnt3a. (C) Relative distribution of various endocytic and vesicular proteins were plotted on the basis of densitometry of individual lanes for each genotype and condition. Rab8a-/- MEFs (red lines) were compared to WT MEFs (blue lines), before (solid lines) and after (dotted lines) Wnt3a stimulation.
